# Risk factors for hypotension and technical complications during single-session double-filtration plasmapheresis: a 15-year retrospective study of 1,022 procedures

**DOI:** 10.1080/0886022X.2026.2667592

**Published:** 2026-06-01

**Authors:** Chenni Gao, Jun Ma, Meng Jing, Zhenhua Yang, Yujia Zhu, Haijin Yu, Jingyuan Xie, Xiaonong Chen, Zijin Chen

**Affiliations:** a Department of Nephrology, School of Medicine, Institute of Nephrology, Shanghai Ruijin Hospital, Shanghai Jiao Tong University, Shanghai, China; b Department of Nephrology, The Second People’s Hospital of Shanxi Province, Taiyuan, China

**Keywords:** Double-filtration plasmapheresis, hyperviscosity syndrome, nafamostat mesylate, hypotension, technical complication

## Abstract

Double-filtration plasmapheresis (DFPP) is increasingly used for immune-mediated and hematologic diseases, yet real-world safety data remain limited. In this 15-year retrospective study, we analyzed 1,022 DFPP sessions performed in 385 patients. The primary outcomes were hypotension and technical complications. Patient demographics, indications, laboratory parameters, treatment prescriptions, anticoagulation regimens, and procedure-related adverse events were collected. Mixed-effects logistic regression models were applied to identify factors associated with hypotension and technical complications. Hyperviscosity syndrome was the most common indication, accounting for 55.3% of all sessions. The mean treatment volume was 3.06 ± 0.41 L, corresponding to 0.95 ± 0.16 times the estimated plasma volume. Hypotension occurred in 15.3% of sessions and technical complications in 7.6%, both demonstrating significant declining trends over time (*p* < 0.05 for trend). Immune thrombotic thrombocytopenic purpura was independently associated with hypotension (OR 11.96, 95% CI 1.94–73.68). Older age modestly increased risk (OR 1.03 per year, 95% CI 1.00–1.06), while treatment year was inversely associated with hypotension (OR 0.77 per year, 95% CI 0.66–0.89). Technical complications were independently associated with use of the PLASAUTO Σ system (OR 8.78, 95% CI 2.76–27.97), use of non-heparin/non-nafamostat anticoagulants (OR 9.42, 95% CI 2.71–32.72), and hyperviscosity syndrome (OR 8.09, 95% CI 1.80–36.29). Among 309 sessions with paired measurements, median immunoglobulin reduction was 29.8% for IgG overall. DFPP demonstrated an acceptable safety profile, with declining complication rates over time. The risk of complications was influenced by underlying disease, patients’ status, and anticoagulation strategy.

## Introduction

Double-filtration plasmapheresis (DFPP) is a semi-selective apheresis technique that removes high-molecular-weight substances, such as immunoglobulins and immune complexes, while largely preserving smaller molecules such as albumin, by using a two-stage membrane filtration process. In the first stage, plasma is separated from blood cells. The plasma then passes through a secondary filter that retains and removes high-molecular-weight pathogens, while allowing low-molecular-weight proteins to return to the patient with the blood cells. DFPP, a membrane-based plasma separation technique, has been widely used in countries such as Japan and Germany [1]. It has become an increasingly important therapeutic modality in a wide range of immune-mediated and hematologic conditions, including hyperviscosity syndrome, systemic lupus erythematosus (SLE), ANCA-associated vasculitis (AAV) [2], anti-glomerular basement membrane (anti-GBM) disease [3], immune thrombotic thrombocytopenic purpura (iTTP) [4], Guillain–Barré syndrome [5], and transplant setting for antibody removal [6]. Therapeutic plasma exchange (TPE) and DFPP are both widely used in nephrological and autoimmune diseases and have demonstrated favorable clinical efficacy.

Compared with TPE, DFPP offers several advantages, including a reduced need for fresh plasma, a lower risk of plasma-related allergic reactions, and greater selectivity in macromolecule removal [7]. Despite its growing use, real-world data on the safety and efficiency of DFPP across diverse patient populations remain limited. In particular, the risk factors associated with procedure-related complications – such as hypotension and technical complication – are poorly characterized.

In this study, we retrospectively reviewed DFPP sessions performed over a 15-year period at a single center. We aimed to describe the clinical practice of DFPP at our institution and to examine factors associated with hypotension and technical complications. We hope that these findings may contribute to the safer and more effective use of DFPP in routine clinical care.

## Methods

### Study participants and study design

**Figure 1.**
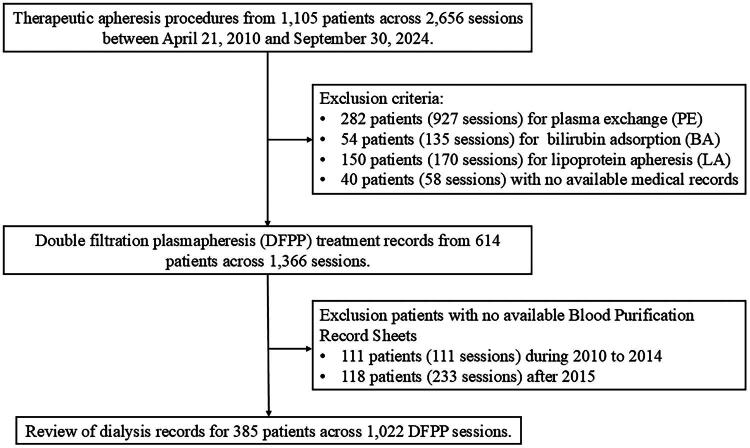
Flowchart illustrating the study design and patient selection process.

Patients who underwent DFPP between 21 April 2010 and 30 September 2024 at the Blood Purification Center of the Department of Nephrology, Ruijin Hospital, Shanghai Jiao Tong University School of Medicine, Shanghai, China were retrospectively reviewed and enrolled. Exclusion criteria were: (1) treatment with single-membrane plasma exchange, lipoprotein apheresis, or bilirubin adsorption; (2) absence of medical records; and (3) lack of blood purification record sheets. A flowchart of the patient selection process is shown in Figure 1. The primary study outcomes were DFPP-related hypotension and technical complications. Hypotension was defined as a blood pressure <90/60 mmHg occurring during treatment or within one hour after completion, accompanied by symptoms such as dizziness or diaphoresis and requiring clinical intervention or resolving with appropriate management. Technical complications included primary transmembrane pressure (TMP1) alarms (>60 mmHg), secondary transmembrane pressure (TMP2) alarms (>300 mmHg), and membrane rupture. All episodes of hypotension and technical complications were identified through retrospective review of treatment logs and medical records.

This retrospective study was approved by the Ethics Committee of Ruijin Hospital, Shanghai Jiao Tong University School of Medicine (Approval No. 2025 [Linlun] No. 512) and was conducted in accordance with the Declaration of Helsinki. Written informed consent was obtained from all participants prior to their inclusion in the study.

### Data collection

Demographic and clinical information, including age, sex, primary disease, and weight was collected. Primary diseases were categorized according to the 2024 American Society for Apheresis (ASFA) guidelines [8], and their indications for DFPP were classified accordingly. Treatment details were extracted from blood purification treatment record sheets, which were reviewed for each session, documenting the date, blood flow rate, prescribed and delivered replacement volumes, treatment duration, and anticoagulation regimen. The anticoagulants used included heparin (unfractionated or low-molecular-weight), nafamostat mesylate, and other agents (fondaparinux or rivaroxaban). Laboratory data included hematocrit (Hct), platelet counts, activated partial thromboplastin time (APTT), and prothrombin time (PT) measured closest to each DFPP session, immunoglobulin levels assessed before and after DFPP, and post-treatment fibrinogen levels. The reduction rate for each immunoglobulin was calculated using the following formula: (pretreatment level − post-treatment level)/pretreatment level × 100%. Sessions with missing data for specific variables were excluded from the corresponding analyses. Medical records were reviewed to document fibrinogen supplementation during DFPP sessions and any bleeding events that occurred during hospitalization.

### DFPP treatment protocol

**Figure 2.**
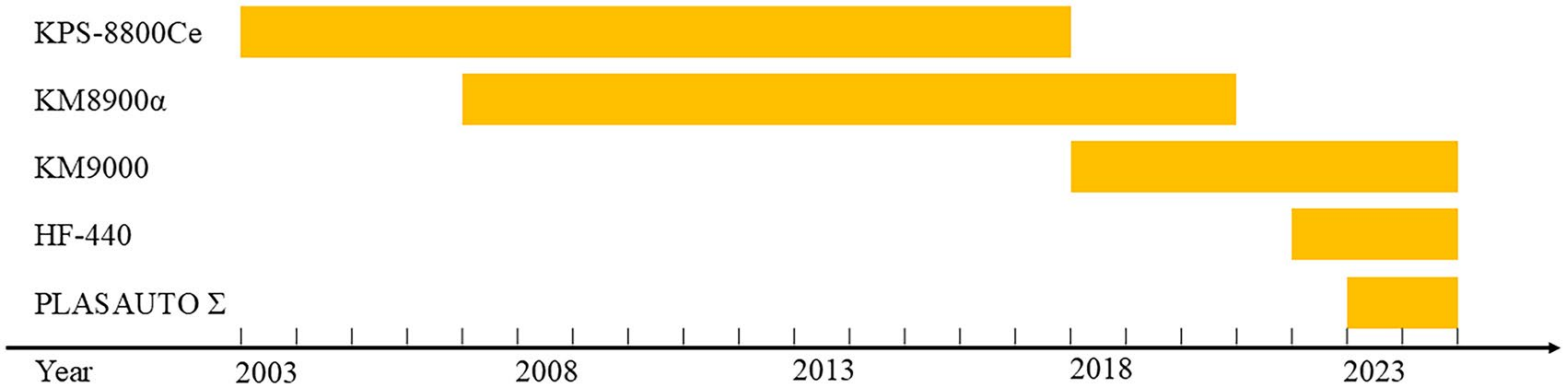
Clinical utilization timeline of DFPP system in our center. This figure illustrates the years of clinical use for each DFPP system at our center. The KPS-8800CE system was in use from 2003 to 2017, KM-8900α from 2007 to 2020, and KM-9000 from 2018 to the present. More recently, the Infomed HF-440 has been used since 2022, and the ACH-Σ (PLASAUTO Σ) system has been in use since 2023.

All DFPP procedures were conducted in accordance with standard operating protocols. Treatments were performed using one of the following blood purification systems: KPS-8800CE, KM-8900α, KM-9000 (Sanyo Electronic Industries Co., Ltd., Okayama, Japan), HF-440 (Infomed SA, Geneva, Switzerland), or ACH-Σ (PLASAUTO Σ) (Asahi Kasei Medical Co., Ltd., Tokyo, Japan). DFPP system selection varied throughout the study period, with the distribution of DFPP equipment usage over time shown in Figure 2. A combination of plasma separation and secondary filtration was used during DFPP procedures. Plasma was first separated using one of the following systems: Plasmaflo OP-08W (Asahi Kasei Medical Co., Ltd., Tokyo, Japan), Plasmacure PE-08 (Asahi Kasei Kuraray Medical Co., Ltd., Tokyo, Japan), or Haemoselect L 0.5 (B. Braun, Melsungen, Germany). Secondary filtration for the selective removal of high-molecular-weight substances was then performed using either the Evaflux (2A20 or 4A40; Kawasumi Laboratories, Inc., Tokyo, Japan) or the Cascadeflo EC (EC-20W or EC-40W; Asahi Kasei Medical Co., Ltd., Tokyo, Japan). For targeted IgM removal, the EC-40W or 4A40 filter was selected; for other molecules, the EC-20W or 2A20 filter was preferred.

DFPP prescriptions and settings were individualized based on the patient’s body weight, Hct, and clinical condition. Blood flow rates were typically set at 90–120 mL/min, and plasma flow rates at 20–24 mL/min. A total of 400–600 mL of 5% albumin was used as the replacement fluid in each session. The treatment volume was expressed as a multiple of the estimated plasma volume (PV). PV was estimated using the following formula: PV = 0.065 × body weight (kg) × (1 − Hct) [9,10]. During DFPP, Fg was routinely monitored only in patients receiving multiple sessions (including those with renal failure), and supplementation was initiated when post-procedure levels fell below 1.5 g/L.

### Statistical analysis

Continuous variables with a normal distribution were presented as mean ± standard deviation, while non-normally distributed data were reported as median (interquartile range). Categorical variables were expressed as counts (percentages). Continuous variables were compared using Student’s *t*-test for normally distributed data and the Wilcoxon rank-sum test for non-normally distributed data. Comparisons among three or more groups were performed using one-way analysis of variance (ANOVA) or the Kruskal–Wallis test, as appropriate. Categorical variables were analyzed using the Chi-square test or Fisher’s exact test. Temporal trends in annual event rates were assessed using weighted linear regression models at the annual level, with calendar year treated as a continuous variable and annual event rates as the dependent variable. Analyses were weighted by the annual number of DFPP sessions. To evaluate factors associated with hypotension and technical complications, mixed-effects logistic regression models were fitted with a random intercept for patient ID to account for within-patient clustering of repeated DFPP sessions. Covariates were selected *a priori* based on clinical relevance and previous literature. The primary multivariable model included age, sex, calendar year, type of DFPP system (KPS-8800CE/KM-8900α/KM-9000, HF-440, or PLASAUTO Σ), type of anticoagulant used during DFPP (heparin, nafamostat mesylate, or others), disease category (hyperviscosity syndromes; kidney diseases including anti-GBM disease or AAV; SLE; iTTP; transplantation-related conditions; and other conditions), and PV multiples. In sensitivity analyses, the fully adjusted models were further adjusted for coagulation-related parameters, including platelet count, APTT, PT, fibrinogen level, and the concomitant use of antiplatelet agents, systemic anticoagulation, and glucocorticoids. All statistical analyses were conducted using STATA version 17.0 (StataCorp LLC, College Station, TX), and a two-sided *p* value < 0.05 was considered statistically significant.

## Results

### Study participants

A total of 385 patients underwent 1,022 DFPP procedures and were included in the analysis. Of these, 209 (54.3%) were male and 176 (45.7%) were female. The mean age was 56.2 ± 15.2 years with range from 12 to 86 years.

According to the 2024 ASFA guidelines, among the treatments performed, 281 cases (73.0%) met category I indications. Categories II and III each accounted for 61 (15.8%) and 43 (11.2%) cases, respectively. The top five indications were hyperviscosity syndrome (*n* = 211, 54.8%), severe SLE (*n* = 25, 6.5%), iTTP (*n* = 22, 5.7%), anti-GBM (*n* = 20, 5.2%), and kidney transplantation (*n* = 20, 5.2%). Distribution of DFPP indications is listed in Table 1.

**Table 1.** Distribution of indications for DFPP treatment according to the 2024 American Society for Apheresis guidelines (*n* = 385).

Indication	Overall (*n* (%))	Category	Recommendation grade
Hyperviscosity in hypergammaglobulinemia	211 (54.8)	I	1B
SLE, severe	25 (6.5)	II	2C
Thrombotic microangiopathy, thrombotic thrombocytopenic purpura	22 (5.7)	I	1A
Anti‐glomerular basement membrane disease	20 (5.2)	I	1B
Transplantation, kidney	20 (5.2)	I	1B
Myeloma cast nephropathy	14 (3.6)	II	2B
Vasculitis, ANCA associated	13 (3.4)	III	1B
Transplantation, hematopoietic stem cell, ABO incompatible	12 (3.1)	II	1B
Idiopathic inflammatory myopathies	7 (1.8)	III	2B
Autoimmune hemolytic anemia, severe	7 (1.8)	III	2C
Cryoglobulinemia	6 (1.6)	II	2A
Acute inflammatory demyelinating polyradiculoneuropathy	5 (1.3)	I	1A
Acute liver failure	4 (1.0)	I	1A
Catastrophic antiphospholipid syndrome	3 (0.8)	I	2C
Graft-versus-host disease	2 (0.5)	II	2B
Hypertriglyceridemic pancreatitis, severe	2 (0.5)	III	1C
Sepsis with multiorgan failure	2 (0.5)	III	2A
Toxic epidermal necrolysis	2 (0.5)	III	2B
Acute fatty liver of pregnancy	1 (0.3)	III	2B
Atopic dermatitis, recalcitrant	1 (0.3)	III	2B
Autoimmune dysautonomia	1 (0.3)	III	2C
Chronic focal encephalitis	1 (0.3)	III	2C
Lipoprotein(a) hyperlipoproteinemia	1 (0.3)	II	1B
Myasthenia gravis	1 (0.3)	I	1B
Neuromyelitis optical spectrum disorder	1 (0.3)	II	1B
Stiff-person syndrome	1 (0.3)	III	2A

During treatment, 270 patients (70.1%) experienced a decline in fibrinogen levels to below 1.8 g/L, and 173 patients (44.9%) received fibrinogen supplementation. Bleeding events occurred in 15 patients (3.9%).

### DFPP sessions overview

A total of 1,022 sessions were performed in 385 patients. The most frequently used system was the KM9000, accounting for 528 sessions (51.7%), followed by the KM8900α (347 sessions, 33.9%), PLASAUTO Σ (95 sessions, 9.3%), KPS8800CE (29 sessions, 2.8%), and HF-440 (23 sessions, 2.3%). The mean treatment duration was 153.8 ± 36.3 min, with an average prescribed treatment volume of 3.06 ± 0.41 L, equivalent to 0.95 ± 0.16 times the estimated PV. Among the sessions, 54 (5.3%) were prematurely terminated, with an actual treatment volume of 3.02 ± 0.46 L achieved. The most common reason for early termination was elevated TMP2 pressure (24 sessions, 44.4%), followed by hypotension (18 sessions, 33.3%), poor catheter flow (five sessions, 9.3%), vomiting (two sessions, 3.7%), tachycardia, profuse sweating, and patient-reported intolerance (each one session, 1.9%).

**Figure 3.**
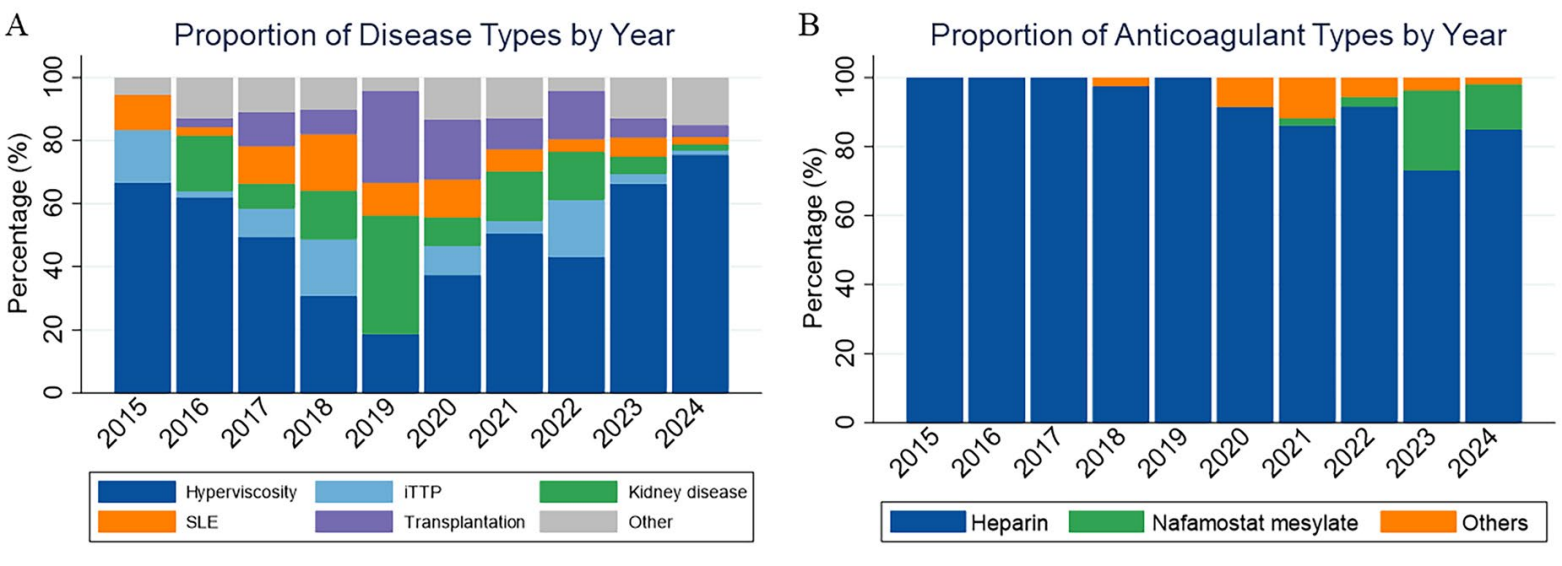
Annual trends in anticoagulant use and disease composition in DFPP sessions (2015–2024). Panel A showed primary disease categories across years, with hyperviscosity syndrome as the leading indication. Panel B showed proportion of different anticoagulants used each year.

Hyperviscosity syndrome was the most common indication for DFPP, accounting for 55.3% of all sessions, with its proportion increasing to 75.5% in 2024 (Figure 3(A)). Kidney diseases comprised 10.2% of sessions, peaking at 37.5% in 2019 but declining to 1.9% in 2024. Transplantation-related indications represented 9.5% overall, with a peak in 2020 (19.0%) and a decrease to 3.8% in 2024. Other indications included iTTP (6.1%), SLE (7.1%), and other conditions (11.8%).

Heparin was the primary anticoagulant used in 89.3% of sessions (913/1,022). Nafamostat mesylate and fondaparinux were used in 6.8% and 3.8% of sessions, respectively. Use of nafamostat mesylate increased substantially after 2021, reaching 23.3% in 2023 and 13.2% in 2024, while fondaparinux was gradually adopted over time (Figure 3(B)). Baseline characteristics according to anticoagulant type are summarized in Table 2, with significant differences observed in age and disease distribution across groups (*p* = 0.035 and *p* = 0.027).

**Table 2.** Baseline characteristics of 1,022 DFPP sessions stratified by anticoagulant type.

	Overall	Heparin	Nafamostat mesylate	Others	*p* Value
*N*	1,022	913	70	39	
Age, years	55.5 ± 15.0	55.0 ± 15.1	59.3 ± 12.8	58.3 ± 15.4	0.035
Male, *n* (%)	569 (55.7)	516 (56.5)	34 (48.6)	19 (48.7)	0.293
Disease category					0.027
Kidney disease	104 (10.2)	91 (10.0)	7 (10.0)	6 (15.4)	
Hyperviscosity	565 (55.3)	515 (56.4)	35 (50)	15 (38.5)	
iTTP	62 (6.1)	51 (5.59)	4 (18.0)	7 (6.1)	
SLE	73 (7.1)	63 (6.9)	6 (8.6)	4 (10.3)	
Transplantation	97 (9.5)	91 (10.0)	4 (5.7)	2 (5.1)	
Other conditions	121 (11.8)	102 (11.2)	14 (20.0)	5 (12.8)	
PV multiples	0.95 ± 0.16	0.95 ± 0.16	0.93 ± 0.14	0.92 ± 0.19	0.448
Platelet count, ×10^9^/L	149 (97, 216)	157 (105, 228)	79.5 (59, 148)	60 (53, 105)	<0.001
APTT, s	30.1 (27.5, 35.4)	30.1 (27.5, 35.7)	31.7 (29.1, 34.7)	31 (24.1, 35.5)	0.105
PT, s	12.7 (11.5, 13.7)	12.7 (11.5, 13.7)	12.8 (11.7, 14.1)	12.2 (12.0, 13.4)	0.353
Fibrinogen, g/L	2.4 (1.8, 3.1)	2.4 (1.9, 3.1)	2.5 (1.5, 3.1)	1.8 (1.3, 2.5)	0.095
Antiplatelet therapy, *n* (%)	48 (4.7)	48 (5.26)	0 (0)	0 (0)	–
Systemic anticoagulation, *n* (%)	283 (27.7)	248 (27.2)	22 (31.4)	13 (33.3)	0.539
Glucocorticoid therapy, *n* (%)	521 (51.3)	458 (50.5)	36 (51.4)	27 (69.2)	0.072

iTTP: immune thrombotic thrombocytopenic purpura; SLE: systemic lupus erythematosus; PV: plasma volume; APTT: activated partial thromboplastin time; PT: prothrombin time.

Data are presented as mean ± SD, median (interquartile range), or *n* (%), as appropriate.

### Temporal trends in DFPP-related complications

**Figure 4.**
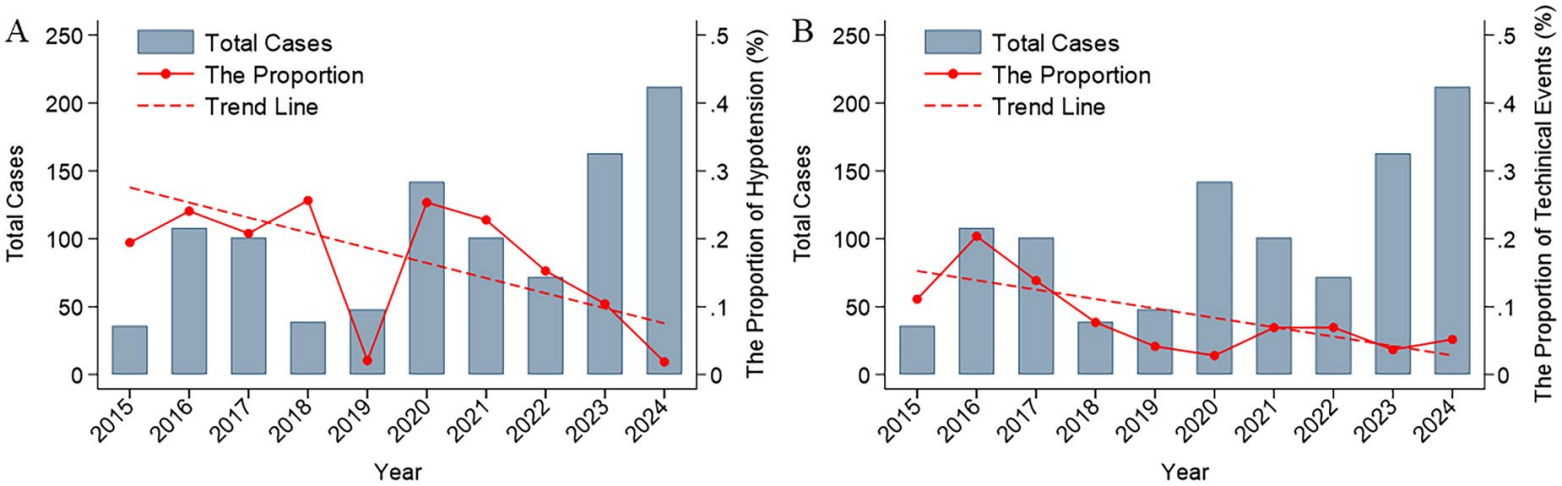
Annual trends of DFPP-related hypotension and technical events. Panel A shows the annual proportion of DFPP-related hypotension. Panel B shows the annual proportion of technical events. Light blue bars represent the number of DFPP sessions. Red dots indicate the proportion of events, and the red dashed line represents the fitted weighted linear regression trend over time.

Overall, hypotension occurred in 15.3% of DFPP sessions, and a total of 78 sessions (7.6%) were complicated by technical events. The annual incidence of hypotension declined from 19.44% in 2015 to 1.89% in 2024. Similarly, the incidence of technical complications decreased from 11.11% in 2015 to 5.19% in 2024. Weighted linear regression analyses confirmed significant decreasing temporal trends in both hypotension (*β* = −0.02 per year, *p* = 0.023, Figure 4(A)) and technical complication rates (*β* = −0.01 per year, *p* = 0.013, Figure 4(B)).

### Risk factors of hypotension during DFPP session

In the mixed-effects logistic regression analysis for hypotension (Table 3), iTTP was independently associated with hypotension in both models. In the primary model, iTTP (OR 13.14, 95% CI 2.20–78.40, *p* = 0.005) and transplantation (OR 5.58, 95% CI 1.06–29.51, *p* = 0.043) were significantly associated with an increased risk of hypotension, whereas treatment year was inversely associated with risk (OR 0.77 per year, 95% CI 0.67–0.89, *p* < 0.001). After additional adjustment for laboratory variables and concomitant medications, the association between iTTP and hypotension remained significant (OR 11.96, 95% CI 1.94–73.68, *p* = 0.007), while transplantation was no longer statistically significant (OR 3.11, 95% CI 0.58–16.69, *p* = 0.186). Higher fibrinogen levels were independently associated with a lower risk (OR 0.52, 95% CI 0.34–0.80, *p* = 0.003), and age with a higher risk (OR 1.03 per year, 95% CI 1.00–1.06, *p* = 0.044). Treatment year remained inversely associated with risk (OR 0.77 per year, 95% CI 0.66–0.89, *p* < 0.001).

**Table 3.** Mixed-effects logistic regression for hypotension.

	Primary analysis			Fully adjusted model		
Variable	OR	95% CI	*p* Value	OR	95% CI	*p* Value
Age, years	1.02	0.996–1.05	0.093	1.03	1.00–1.06	0.044
Male (vs. female)	0.65	0.31–1.38	0.264	0.81	0.39–1.69	0.571
Year	0.77	0.67–0.89	**<0.001**	0.77	0.66–0.89	**<0.001**
Type of DFPP system						
KPS-8800CE/KM-8900α/KM-9000	Ref.			Ref.		
HF-400	0.56	0.07–4.57	0.590	0.66	0.08–5.14	0.690
PLASAUTO Σ	1.05	0.31–3.57	0.933	0.95	0.27–3.33	0.936
Anticoagulant type						
Heparin	Ref.			Ref.		
Nafamostat mesylate	0.18	0.02–1.89	0.153	0.21	0.02–2.11	0.184
Others	2.09	0.61–7.22	0.243	2.15	0.63–7.35	0.222
Primary disease categories						
Kidney disease	Ref.			Ref.		
Hyperviscosity	2.45	0.69–8.66	0.163	1.980	0.50–7.86	0.330
iTTP	13.14	2.20–78.40	**0.005**	11.960	1.94–73.68	**0.007**
SLE	1.16	0.16–8.56	0.881	1.13	0.15–8.24	0.906
Transplantation	5.58	1.06–29.51	**0.043**	3.11	0.58–16.69	0.186
Other conditions	1.92	0.38–9.59	0.427	2.03	0.42–9.85	0.379
PV multiples	1.45	0.19–10.89	0.720	1.08	0.14–8.36	0.939
Platelet count, ×10^9^/L		–		1.00	1.00–1.01	0.071
APTT, s		–		0.99	0.95–1.04	0.704
PT, s		–		1.00	0.80–1.25	0.976
Fibrinogen, g/L		–		0.52	0.34–0.80	**0.003**
Antiplatelet therapy, *n* (%)		–		0.49	0.06–3.80	0.497
Systemic anticoagulation, *n* (%)		–		1.21	0.53–2.75	0.648
Glucocorticoid therapy, *n* (%)		–		0.64	0.27–1.52	0.314

OR: odds ratio; CI: confidence interval; iTTP: immune thrombotic thrombocytopenic purpura; SLE: systemic lupus erythematosus; PV: plasma volume; APTT: activated partial thromboplastin time; PT: prothrombin time.

The intraclass correlation coefficient (ICC) was 0.50 for the basic model and 0.44 for the fully adjusted model.

The bold *p* values represented statistically significant.

### Risk factors of technical complications during DFPP session

In the primary analysis (Table 4), use of the PLASAUTO Σ system (OR 9.29, 95% CI 2.77–31.15, *p* < 0.001), use of other anticoagulants (OR 8.70, 95% CI 2.27–33.43, *p* = 0.002), and hyperviscosity syndrome (OR 6.46, 95% CI 1.48–28.15, *p* = 0.013) were significantly associated with an increased risk of technical complications. Treatment year was inversely associated with risk (OR 0.70 per year, 95% CI 0.61–0.82, *p* < 0.001). In the fully adjusted model, increasing age was associated with a higher risk of technical complications (OR 1.03 per year, 95% CI 1.00–1.06, *p* = 0.047), whereas treatment year remained inversely associated with risk (OR 0.71 per year, 95% CI 0.61–0.82, *p* < 0.001). Use of the PLASAUTO Σ system (OR 8.78, 95% CI 2.76–27.97, *p* < 0.001), use of other anticoagulants (OR 9.42, 95% CI 2.71–32.72, *p* < 0.001), and hyperviscosity syndrome (OR 8.09, 95% CI 1.80–36.29, *p* = 0.006) remained significantly associated with increased risk. PV multiples was associated with a lower risk (OR 0.07, 95% CI 0.008–0.55, *p* = 0.012), and platelet count showed a small but statistically significant positive association (OR 1.004 per unit, 95% CI 1.000–1.009, *p* = 0.042). The patient-level random effect was not statistically significant in the fully adjusted model (likelihood ratio test *p* = 0.29), and the intraclass correlation coefficient decreased to 0.08.

**Table 4.** Mixed-effects logistic regression for technical complication.

	Primary analysis			Fully adjusted model		
Variable	OR	95% CI	*p* Value	OR	95% CI	*p* Value
Age, years	1.01	0.98–1.05	0.386	1.03	1.00–1.06	**0.047**
Male (vs. female)	1.80	0.82–3.96	0.143	1.79	0.86–3.74	0.122
Year	0.70	0.61–0.82	**<0.001**	0.71	0.61–0.82	**<0.001**
Type of DFPP system						
KPS-8800CE/KM-8900α/KM-9000	Ref.			Ref.		
HF-400	4.47	0.72–27.67	0.108	4.08	0.73–22.86	0.109
PLASAUTO Σ	9.29	2.77–31.15	**<0.001**	8.78	2.76–27.97	**<0.001**
Anticoagulant type						
Heparin	Ref.			Ref.		
Nafamostat mesylate	0.31	0.03–3.00	0.312	0.42	0.05–3.68	0.436
Others	8.70	2.27–33.43	**0.002**	9.42	2.71–32.72	**<0.001**
Primary disease categories						
Kidney disease	Ref.			Ref.		
Hyperviscosity	6.46	1.48–28.15	**0.013**	8.09	1.80–36.29	**0.006**
iTTP	1.15	0.09–15.66	0.914	1.80	0.14–22.94	0.65
SLE	2.48	0.27–23.20	0.426	3.58	0.42–30.24	0.242
Transplantation		–			–	
Other conditions	1.53	0.24–9.71	0.654	2.00	0.35–11.46	0.437
PV multiples	0.09	0.01–0.85	**0.035**	0.07	0.008–0.55	**0.012**
Platelet count, ×10^9^/L		–		1.004	1.000–1.009	**0.042**
APTT, s		–		0.98	0.93–1.02	0.335
PT, s		–		1.04	0.86–1.26	0.676
Fibrinogen, g/L		–		0.85	0.58–1.25	0.403
Antiplatelet therapy, *n* (%)		–		0.43	0.04–5.27	0.509
Systemic anticoagulation, *n* (%)		–		1.24	0.59–2.61	0.573
Glucocorticoid therapy, *n* (%)		–		1.46	0.66–3.24	0.345

OR: odds ratio; CI: confidence interval; iTTP: immune thrombotic thrombocytopenic purpura; SLE: systemic lupus erythematosus; PV: plasma volume; APTT: activated partial thromboplastin time; PT: prothrombin time.

The intraclass correlation coefficient (ICC) was 0.24 for the basic model and 0.08 for the fully adjusted model.

The bold *p* values represented statistically significant.

### Efficacy of DFPP in immunoglobulin removal

**Figure 5.**
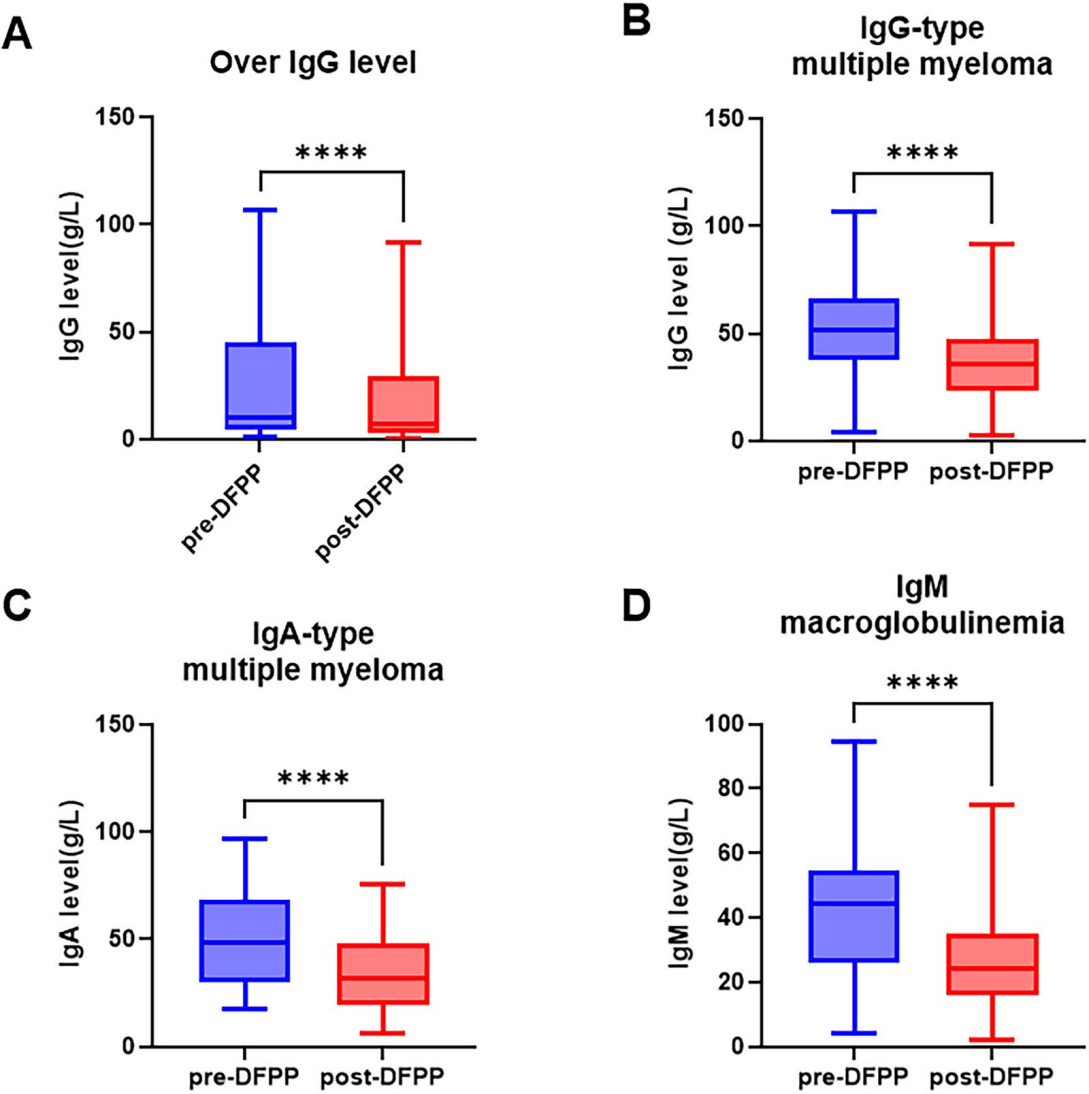
Changes in immunoglobulin levels before and after DFPP treatment. The overall serum levels of IgG (*n* = 309, A) before and after DFPP treatment. Subgroup analyses include IgG levels in IgG-type multiple myeloma (*n* = 125, B), IgA levels in IgA-type multiple myeloma (*n* = 53, C), and IgM levels in Waldenström macroglobulinemia (*n* = 48, D). Statistical comparisons were performed using the Wilcoxon matched-pairs signed rank test. ****p < 0.0001.

Totally, 309 DFPP sessions with available paired immunoglobulin measurements, changes in immunoglobulin levels before and after treatment are illustrated in Figure 5. The overall reductions were as follows: IgG decreased by a median of 2.88 g/L (IQR, 1.09–10.87), representing a 29.8% (IQR, 19.9–39.2%) reduction.

When stratified by primary disease, 125 DFPP sessions in patients with IgG-type multiple myeloma showed a median IgG reduction of 15.2 g/L (IQR, 7.2–20.4), with a 30.2% (IQR, 20.9–39.6%) decline. For 53 sessions in patients with IgA-type MM, IgA decreased by 13.7 g/L (IQR, 9.2–20.3), corresponding to a 32.1% (IQR, 24.3–42.8%) reduction. In 48 sessions for Waldenström’s macroglobulinemia (IgM type), IgM levels decreased by 16.0 g/L (IQR, 7.1–23.9), with a 42.8% (IQR, 31.3–50.2%) reduction. In 54 sessions for other diseases, IgG decreased by 3.1 g/L (IQR, 1.5–5.0), with a 34.8% (IQR, 28.6–44.6%) reduction.

## Discussion

Our study represents one of the larger single-center experiences with DFPP in a real-world clinical setting and offers additional insight into its safety and practical use across a range of disease conditions. The indications at our center were largely aligned with ASFA recommendations, mainly including hyperviscosity syndromes and autoimmune diseases. In this cohort, hypotension and technical complications occurred in 15.3% and 7.6% of sessions, respectively. Encouragingly, the incidence of both complications declined significantly over time, likely reflecting growing experience and refinements in procedural management. In multivariable analyses, iTTP was associated with a higher risk of hypotension, whereas hyperviscosity syndromes and the use of non-heparin anticoagulants (other than nafamostat mesylate) were associated with an increased risk of technical complications. Together, these findings help to better characterize the safety profile of DFPP and may assist in identifying patients at higher risk of procedure-related adverse events.

DFPP was generally well tolerated in our cohort, and the incidence of complication was comparable to that reported in previous studies (14.0% [11] and 7.6% [1]). Hypotension was the most common adverse event observed. Several mechanisms may contribute to treatment-related hypotension during DFPP, including intravascular volume shifts and rapid plasma removal. Notably, patients with iTTP were more likely to develop hypotension. This may be related to the systemic inflammatory state, endothelial injury, and microvascular instability characteristic of iTTP, which could increase susceptibility to hemodynamic fluctuations during extracorporeal therapy. Higher fibrinogen levels appeared to confer a protective effect, whereas older age was associated with a modestly increased risk. Together, these findings suggest that both coagulation status and patient-specific factors influence hemodynamic tolerance during DFPP. Bradykinin-mediated mechanisms may also play a role. The Plasmaflo OP-05W membrane has been reported to increase bradykinin levels, and angiotensin-converting enzyme inhibitors (ACEIs) are therefore generally withheld prior to treatment [12]. Given that only eight patients were treated with ACEIs, we were unable to adequately evaluate the relationship between ACEI use and the risk of hypotension.

Technical complications are also common during DFPP sessions. Hyperviscosity syndrome was associated with an increased risk of technical complications, which may be related to elevated plasma viscosity predisposing to filter clotting and rising transmembrane pressure (TPM) during the procedure. In addition, the use of anticoagulants other than heparin or nafamostat mesylate was also associated with a higher risk of technical complications. Age and coagulation-related parameters were also linked to technical events. However, the choice of anticoagulant is often determined by patients’ baseline coagulation status and overall clinical condition. Therefore, individualized selection and timely adjustment of anticoagulation strategies are essential to optimize circuit stability and reduce procedure-related technical events. We also observed significant variation in technical complication rates across DFPP systems. The PLASAUTO Σ system was associated with a higher risk of technical complications; however, the mechanisms underlying this association are uncertain. Differences in system configuration, circuit design, or operator familiarity may partially explain this finding. Further studies are warranted to clarify whether this reflects system-specific characteristics or residual confounding.

Our study also observed the use of different anticoagulants during DFPP procedures. Although a recent report involving nearly 1,000 membrane-based plasma exchange sessions using the NxStage machine showed that only 7% required anticoagulation [11], all DFPP sessions in our center were performed with anticoagulation. Heparin remained the most commonly used agent and demonstrated a balanced safety profile. For patients with thrombocytopenia or high bleeding risk, fondaparinux and nafamostat mesylate are viable alternatives, although their application in DFPP is rarely reported in the literature. In pediatric plasma exchange, nafamostat mesylate has demonstrated anticoagulation efficacy comparable to citrate, with fewer metabolic and adverse effects [13]. In our analysis, nafamostat mesylate was associated with a risk of technical complications similar to that observed with heparin, whereas other anticoagulants, including fondaparinux and rivaroxaban, were linked to a higher risk. These findings may reflect differences in anticoagulation efficacy within the extracorporeal circuit. However, unmeasured clinical factors may also have contributed to the observed associations.

With respect to treatment prescription, the prescribed treatment volume in our retrospective cohort was lower than guideline recommendations of 1.0–1.5 times the estimated PV. The mean volume achieved was 3.06 ± 0.41 L (0.95 ± 0.16 times PV), which is also below the approximately 3.6 L commonly reported in conventional TPE [11]. Although PV was routinely estimated using Hct-based formulas, clinicians tended to adopt a more conservative dosing strategy in our center. Interestingly, higher prescribed PV multiples were associated with a lower risk of technical complications. This finding may reflect clinical decision-making rather than a direct protective effect of treatment intensity. Clinicians may be more inclined to prescribe a full or higher treatment dose in patients who are hemodynamically stable and considered suitable for longer procedures, whereas reduced doses may be selected for patients perceived to be at higher procedural risk. In this context, higher PV multiples may serve as a marker of baseline stability rather than an independent protective factor. Therefore, this association should be interpreted cautiously, as it may partly represent confounding by indication.

Regarding treatment efficacy, DFPP was effective across a broad spectrum of indications. Previous studies have shown that exchanging one estimated PV reduces pretreatment immunoglobulin levels by approximately 63%, and exchanging 1.43 PV results in a 75% reduction [14,15]. In our study, the average PV exchanged was 0.95 times the estimated PV, leading to an overall IgG reduction of 29.8% – substantially lower than the theoretical expectation. This discrepancy may be related to lower exchange volume, ongoing antibody production from the underlying disease and IgG redistribution. Among patients with different MM subtypes, a single DFPP session resulted in similar removal rates for IgA and IgG (30.2% vs. 32.1%). In patients with Waldenström’s macroglobulinemia, the median reduction in IgM was 42.8%, which is consistent with published predictive formulas estimating a 44.9% decrease [16–18]. When comparing DFPP and TPE for treating Waldenström macroglobulinemia, previous reports showed serum IgM reduction rates of 42% with TPE and 27% with DFPP at comparable processed PV [19]. Our findings suggest that DFPP can achieve IgM reduction rates comparable to those of TPE.

There are several strengths to this study. First, it represents the largest summary of DFPP experience in a single center to date, capturing both safety and efficacy outcomes. Second, the patient population reflects a real-world, tertiary hospital setting in China, enabling evaluation across a variety of underlying conditions. Most importantly, the availability of detailed dialysis records allowed for accurate identification of treatment parameters and complications, offering critical insights into optimizing DFPP protocols. Nonetheless, this study has limitations. As a retrospective, single-center analysis, the findings may not be generalizable to other settings. The reliance on historical medical records and blood purification treatment sheets introduces the possibility of incomplete or missing data. Additionally, baseline disease severity (e.g., blood viscosity, and hemodynamic status) may not have been fully accounted for in our analysis. Complications such as hypotension may be influenced by a combination of patient-specific factors and treatment settings, making it difficult to isolate the effects of DFPP technology alone. While this study evaluated the procedural efficacy of a single DFPP session based on immunoglobulin reduction, it did not assess long-term clinical outcomes such as renal recovery or mortality. Nevertheless, this study included a large cohort of DFPP treatment sessions, providing valuable insights for optimizing DFPP procedures and prescriptions.

In summary, DFPP was generally safe and well tolerated in our cohort, with adverse event rates comparable to those reported previously. Hypotension and technical complications appeared to be influenced not only by procedural factors but also by underlying disease characteristics and patient-specific conditions. Differences in anticoagulation strategies may further affect circuit performance and treatment tolerance. These findings highlight the importance of individualized risk assessment and tailored procedural management in optimizing DFPP safety. Prospective studies are warranted to further clarify optimal anticoagulation approaches and identify strategies to minimize complications in high-risk populations.

## Data Availability

The datasets used and/or analyzed during the current study are available from the corresponding author on reasonable request.
